# Understanding how emotional-social intelligence influences voice behavior and satisfaction through self-efficacy in nursing students

**DOI:** 10.1186/s12912-026-04298-4

**Published:** 2026-02-19

**Authors:** Manal Saleh Moustafa Salehs, Azza Abdeldayem Ata, Sahar Rafdan Alshahrani, Sahar Hamdy El-Sayed, Reem Hashem Fathy, Hanan Awad M. Elmashad, Aisha Elsayed-El Araby Abdelwahid

**Affiliations:** 1 Nursing Administration, Faculty of Nursing, Zagazig University, Zagazig, Egypt; 2 Nursing Administration and Education Department, College of Nursing, King Khalid University, Abha, Saudi Arabia; 3 Faculty of Dentistry, Zagazig University, Zagazig, Egypt; 4 Maternal and Pediatric Nursing Department, College of Nursing, King Khalid University, Abha, Saudi Arabia

**Keywords:** Emotional-social intelligence, Voice behaviors, Satisfaction, Self-efficacy, Nursing students

## Abstract

**Aim:**

This study investigates the influence of emotional-social intelligence on nursing students’ voice behaviors and satisfaction, with self-efficacy serving as a mediating factor.

**Background:**

Nursing is inherently an emotionally and socially demanding profession that requires effective communication, emotional attunement, and proactive engagement. Emotional-social intelligence has been shown to significantly foster intrapersonal resources (e.g., emotion regulation, social confidence), strengthening interpersonal relationships, reducing stress, and improving professional satisfaction.

**Subjects and methods:**

A descriptive correlational cross-sectional study was conducted among nursing students in the Faculty of Nursing, Zagazig University, Egypt. Four standardized questionnaires were used to examine nursing students’ emotional-social intelligence, voice behavior, satisfaction, and self-efficacy; 381 nursing students were surveyed. The study’s hypothetical model was examined using AMOS structural equation modeling (SEM).

**Results:**

Emotional-social intelligence had a significant direct effect on self-efficacy, voice behavior, and satisfaction among the studied students. Similarly, self-efficacy partially mediates the link between emotional-social intelligence and students’ voice behavior. Likewise, self-efficacy partially mediates the link between emotional-social intelligence and students’ satisfaction.

**Conclusions:**

Students with greater emotional-social intelligence are more assertive in expressing ideas, which enriches their satisfaction with learning and clinical capabilities. These results highlight the significance of nurturing emotional-social intelligence in nursing education to improve their positive communication and professional growth.

**Clinical trial number:**

Not applicable.

## Introduction

Nursing education aims to prepare students to become competent professionals by fostering awareness, responsibility, and appropriate professional behaviors [1]. As healthcare systems grow more complex and patient expectations increase, nursing students are expected to develop not only clinical knowledge but also independence, adaptability, and strong interpersonal skills [2]. In this context, emotional social intelligence (ESI), the ability to recognize and regulate emotions and apply this understanding to support stress management, clear communication, empathy, and constructive conflict resolution, plays a vital role [3].

ESI strengthens students’ confidence to express their opinions, identify concerns, and offer constructive suggestions, thereby promoting positive voice behavior. It is defined as individuals’ intentional efforts to identify existing or potential issues and propose solutions that improve organizational functioning [4]. Engaging in voice behavior reflects a sense of ownership and commitment, which supports effective decision-making. For nurses, speaking up about workplace concerns enhances involvement in decision-making, increases empowerment, and improves overall satisfaction [5].

In higher education, student satisfaction is commonly defined as the personal judgment, students make when comparing their academic expectations with the actual quality of their educational experience [6]. It is shaped by ongoing interactions among learners, faculty, and the broader institutional environment [7]. The literature highlights four key determinants of student satisfaction: curriculum design, teaching quality, opportunities for social engagement, and the overall learning context [8].

Another important psychological factor in nursing education is self-efficacy, which reflects students’ beliefs in their capacity to complete tasks and achieve desired outcomes. High self-efficacy fosters resilience, persistence, and effective problem-solving [9].

## Literature review and hypothesis development

The concept of emotional intelligence (EI) was first introduced by Salovey and Mayer [10], who defined it as the ability to recognize, understand, and regulate emotions in ways that allow emotions to guide behavior constructively. Their ability-based model emphasized processing emotional information to support decision-making. Goleman [11] expanded EI by identifying five components: self-awareness, self-regulation, motivation, empathy, and social skills; linking EI to performance and interpersonal effectiveness.

Later, Bar-On [12] suggested a broader framework that incorporates emotional, social, and personality factors influencing daily functioning. This model aligns closely with the interpersonal and intrapersonal demands of nursing education, making it particularly relevant for understanding the social and emotional competencies required for effective patient care and professional communication. Cumulatively, these perspectives have contributed to three primary EI models: the ability model, Goleman’s competency model, and Bar-On’s emotional–social model [13].

According to Bar-On [14], emotional–social intelligence consists of interconnected competencies that influence how individuals understand and express themselves, relate to others, and manage everyday demands. These competencies are organized into five dimensions: intrapersonal skills, interpersonal skills, stress management, adaptability, and general mood.

Empirical evidence reveals that higher levels of ESI augment adaptability in challenging situations, develop social functioning, reinforce emotional self-regulation, and support both individual and collaborative relationships, ultimately contributing to effective leadership and professional performance [15]. Within nursing education, greater ESI, specifically self-awareness, interpersonal skills, and stress tolerance, has been connected with enhanced confidence, communication capabilities, and social responsibility. These aptitudes empower students to express their opinions, share ideas, and participate actively in learning environments, which are central elements of voice behavior [16].

Voice behavior was first studied in nursing as a proactive communication process through which nurses share related information, suggest innovations, and report risks to enrich patient safety and organizational outcomes [17]. Positive voice behavior also contributes to a supportive psychosocial environment, promotes efficient use of resources, and supports organizational sustainability. At the individual level, it decreases stress, develops the work climate, and augments performance [18]. Accordingly, the following hypothesis is proposed:

### H1

Emotional social intelligence has a positive and significant effect on nursing students’ voice behavior.

Recent studies further indicate that individuals who express constructive ideas and concerns tend to be more engaged and receive greater institutional and leadership support, which contributes to higher satisfaction levels [19]. In higher education settings, satisfaction emerges from dynamic interactions among students, faculty, and the learning environment [7]. Among nursing students, satisfaction enhances self-confidence and self-efficacy, both of which are essential for academic progression and clinical competence [8]. Therefore, the following hypotheses are proposed:

### H2

Emotional social intelligence has a positive and significant effect on nursing students’ satisfaction.

### H3

Emotional social intelligence has a positive and significant effect on nursing students’ self-efficacy.

Previous research in nursing and health education consistently reports positive associations between emotional intelligence and self-efficacy, with students demonstrating stronger emotional competencies reporting greater confidence in task performance and challenge management [20]. Self-efficacy refers to individuals’ beliefs in their capability to manage life events, including academic and professional demands. According to Bandura [21], self-efficacy develops through mastery experiences, vicarious experiences, verbal persuasion, and physiological and emotional states [22].

Self-efficacy not only regulates behavior and skill application but also supports individuals to utilize their capabilities effectively. Studies have shown that higher self-efficacy among nursing students is connected with higher satisfaction and academic success [23]. From a social cognitive perspective, individuals’ beliefs about their capabilities strongly influence their behavioral choices and responses to challenges [21]. ESI enhances self-efficacy by facilitating emotional regulation, constructive interpretation of feedback, and persistence under stress [24].

Consequently, self-efficacy functions as a psychological mechanism linking ESI with key educational and behavioral outcomes. Nursing students with strong emotional competencies tend to communicate confidently, cope effectively under pressure, and engage constructively with others, thereby enhancing voice behavior and satisfaction [25]. Literature on speaking up further indicates that perceived confidence and competence are critical predictors of voice behavior. When nursing students possess high self-efficacy, they are better equipped to manage stress, perform effectively in demanding situations, and experience higher satisfaction, supporting its mediating role [26]. Thus, the following hypotheses are proposed:

### H4

Self-efficacy mediates the relationship between emotional social intelligence and nursing students’ voice behavior.

### H5

Self-efficacy mediates the relationship between emotional social intelligence and nursing students’ satisfaction.

Despite growing interest in emotional intelligence, limited research has examined the role of emotional–social intelligence in fostering voice behavior among nursing students. Moreover, although self-efficacy is well established as a predictor of academic success, its mediating role in the relationships between ESI, proactive communication, and student satisfaction remains underexplored. Existing studies often examine these constructs in isolation rather than within an integrated framework. Therefore, the present study addresses this gap by developing and testing a conceptual model that examines the interrelationships among emotional–social intelligence, voice behavior, student satisfaction, and self-efficacy among nursing students.

**Fig. 1:**
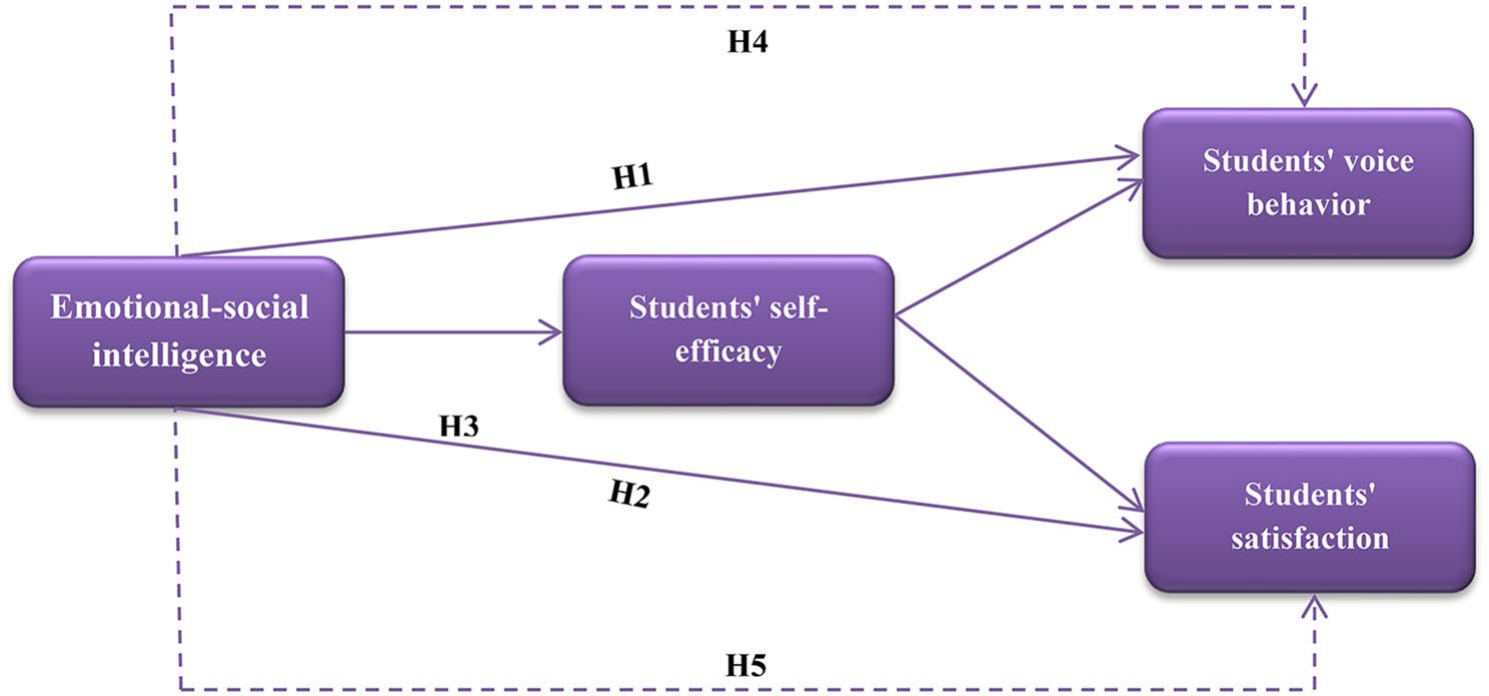
The proposed conceptual model of the study

Based on the hypotheses outlined above, the proposed conceptual research model is presented in Fig. 1.

### The study objective

The purpose of this study is to investigate the influence of emotional-social intelligence on nursing students’ voice behaviors and satisfaction, with self-efficacy serving as a mediating factor.

## Methodology

### Study design

A descriptive correlational cross-sectional design was chosen, which integrates both descriptive and correlational approaches. This design is to describe variables and examine relationships among these variables [27].

### Study setting and participants

This study was conducted at the Faculty of Nursing, Zagazig University, Egypt, during the 2024/2025 academic year. The target population consisted of 1,436 undergraduate nursing students enrolled across four academic levels: first year (*n* = 396), second year (*n* = 378), third year (*n* = 335), and fourth year (*n* = 327). Eligible participants were regular students registered in the undergraduate program who voluntarily agreed to participate. Exclusion criteria included students repeating the academic year, those enrolled in bridging programs (e.g., the Intensive Nursing Program or Special Nursing Program), and students absent during the specific days of data collection.

### Sampling process and sample size

To ensure the sample accurately represented the student population distribution, a stratified proportionate random sampling technique was employed. The minimum sample size was calculated using the finite population formula: n = (N × z² × p̂ (1 − p̂)) / (ε² × (*N* − 1) + z² × p̂ (1 − p̂)) [28]. Based on the total population (*N* = 1,436), a 95% confidence level (z = 1.96), a 5% margin of error (ε = 0.05), and a conservative estimated proportion of 50% (p̂=0.5), the required minimum sample size was determined to be 303 students.

### Substituting the values

$$\begin{aligned}\:\mathrm{n}\:=\frac{\:\left(1436\:{*}\:\right(1.96)^{2}{*}\:0.5\:(1\--0.5\left)\right)\:}{\:\left(\right(0.05)^{2}{*}\:(1436\--1)+\:(1.96)^{2}{*}\:0.5\:(1\--0.5\left)\right)}\end{aligned}$$

$$\begin{aligned}\:\mathrm{n}\:=\frac{\:\left(1436\:\mathrm{*}\:3.8416\:\mathrm{*}\:0.25\right)\:}{\:(0.0025\:\mathrm{*}\:1435\hspace{0.17em}+\hspace{0.17em}0.9604)}\end{aligned}$$

$$\:\mathrm{n}\:=\frac{\left(1377.76\right)}{(3.5875+0.9604)}$$

$$\:\mathrm{n}\:=\frac{\left(1377.76\right)}{\left(4.5479\right)}$$

$$\:\mathrm{n}\hspace{0.17em}\approx\:\hspace{0.17em}303$$

**Fig. 2:**
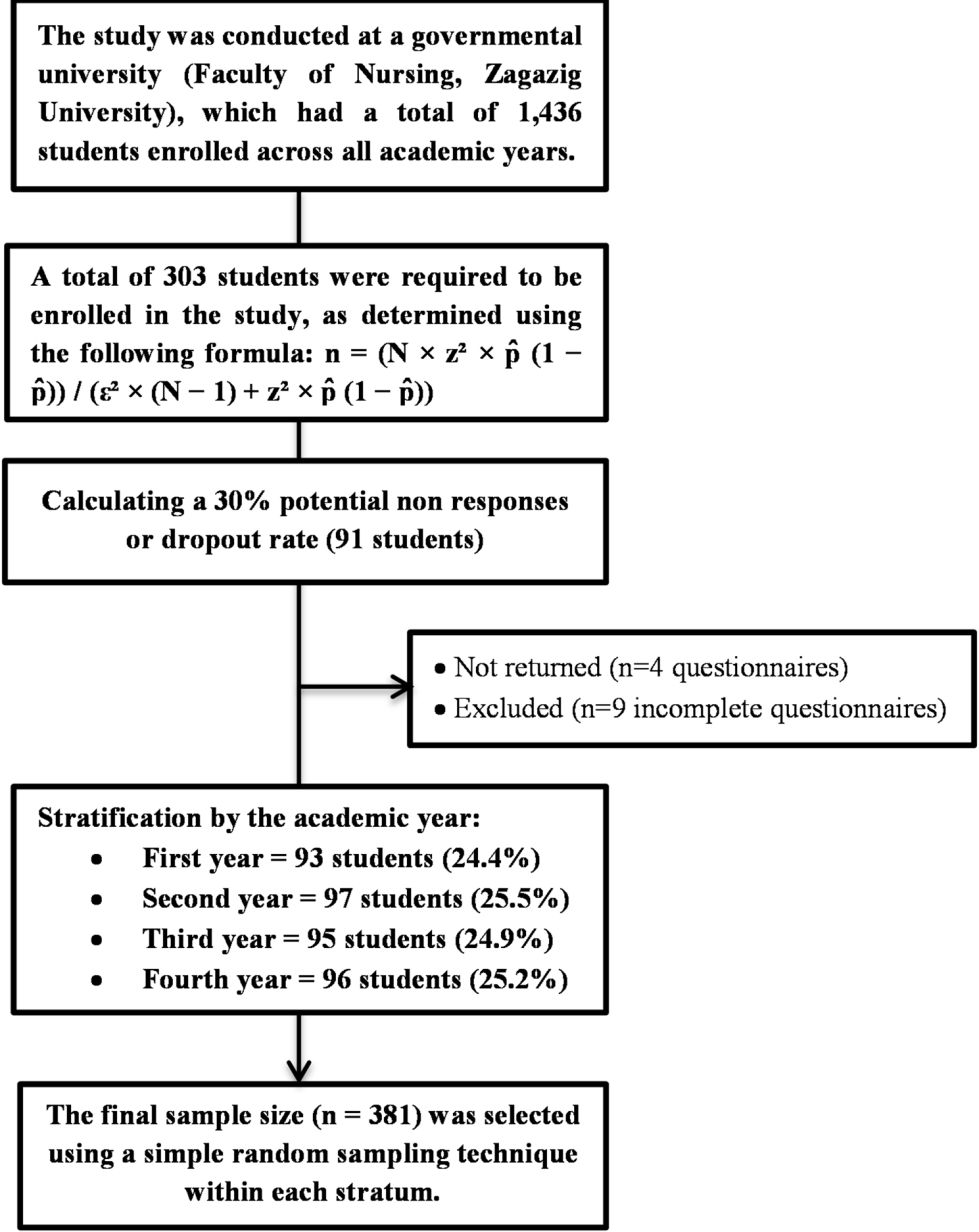
Flow diagram of the sampling process

To account for potential nonresponses and to ensure robust statistical power for the Structural Equation Modeling (SEM) analyses, the target sample was increased by approximately 30%, resulting in 394 distributed surveys. Of the 394 distributed surveys, 390 were returned. After screening for completeness, nine invalid responses were excluded, resulting in a final sample of 381 participants and an effective response rate of 96.7%. The sample was allocated proportionally to each academic year (stratum) based on its size relative to the total population. Within each stratum, participants were selected using simple random sampling via a lottery method using class registration lists, as illustrated in Fig. 2 [29].

### Instruments used in the study

This study utilized four standardized measurement instruments, originally developed in English and subsequently translated and administered into Arabic, in addition to a demographic section, to collect data and perform analyses.

### Tool 1. Emotional–social intelligence scale

It was originally developed by [30] as a self-report measure intended to assess emotional-social intelligence for nursing students, with its validity subsequently confirmed by [16]. This scale is composed of six main components, each comprising specific competencies relevant to undergraduate nursing students. The intrapersonal component includes 16 competencies organized into five subscales: Self-esteem, self-awareness, assertiveness, independence, and self-actualization. The interpersonal component consists of 13 competencies grouped into three subscales: Empathy, social responsibility, and interpersonal relationships. The core nursing skills component contains 12 competencies. The adaptability component encompasses nine competencies divided into three subscales: Reality testing, flexibility, and problem solving. The stress management component is represented by eight items grouped into two subscales: Stress tolerance and impulse control. Finally, the general mood component is measured through seven items grouped into two subscales: Optimism and happiness.

The instrument uses a five-point Likert scale, with item response scores ranging from 1 (not true for me) to 5 (true for me). The score of the items was summed up and divided by the number of items, giving a mean score, then multiplied by 100, and converted into a mean percent. The probable scores range from 65 to 325. The total score for the scale was categorized into ‘’ unsatisfactory ‘’ which had a score less than 60% and ‘’satisfactory’’ which had a score of 60% or more. The Cronbach’s alpha for the entire ESI was reported by the developed author to be 0.96, while the reliability of the tool in this study ranged from 0.84 to 0.98, resulting in α = 0.95 for the total instrument.

### Tool 2. Students’ self-efficacy questionnaire

The students’ self-efficacy questionnaire was developed by [31] to evaluate nursing students’ levels of self-efficacy. The instrument consists of five items, such as “I expect to do as well as or better than other students in the nursing courses.” The students’ self-efficacy questions were measured using a 4-point Likert scale (1–4), where 1 refers to never and 4 refers to always. The score of the items was summed up and divided by the number of items, giving a mean score, then multiplied by 100, and converted into a mean percent. The probable scores range from 5 to 20. The scale’s scoring system is categorized into the following three categories: low self-efficacy (< 60%), moderate self-efficacy (60 – < 80%), and high self-efficacy (≥ 80%). The higher the score means the greater the student’s self-efficacy according to [31]. In the present study, the overall Cronbach’s alpha for the scale was 0.95.

### Tool 3. Voice behavior scale

We used the promotive and prohibitive voice scale, developed by [32], to measure students’ voice. This 10-item instrument distinguishes between two dimensions: Promotive voice, which refers to suggesting improvements in work practices, and prohibitive voice, which relates to raising concerns about harmful practices or behaviors. The Dutch version of the scale was psychometrically validated by [33], providing strong evidence of its validity. Responses were measured on a five-point Likert scale ranging from 1 (never) to 5 (always). The score of the items was summed up and divided by the number of items, giving a mean score, then multiplied by 100, and converted into a mean percent. The probable scores range from 10 to 50. The scale’s scoring system is determined based on cutoff points into the following three categories: Low level of voice behavior < 60%, moderate level 60 – < 80%, and high level of voice behavior ≥ 80%. In the present study, the overall Cronbach’s alpha for the scale was 0.97, with values for the subscales ranging from 0.96 to 0.97.

### Tool 4. Students’ satisfaction questionnaire

This questionnaire was developed by [34] to measure the level of satisfaction among nursing students. It consists of 39 items divided into three main categories. The first category, satisfaction with faculty staff, support staff, and employees, comprises 20 items (e.g., *Faculty staff is polite and courteous*). The second category, satisfaction with the physical environment, includes nine items (e.g., *the library has an extensive collection of available books and periodicals*). The third category, satisfaction with the learning courses provided by the faculty, consists of 10 items (e.g., *As a result of attending the learning courses*,* I have acquired a broad general education in different fields*).

The students’ satisfaction questions were measured using a 4-point Likert scale (1–4), where 1 refers to never and 4 refers to always. The score of the items was summed up and divided by the number of items, giving a mean score, then multiplied by 100, and converted into a mean percent. The probable scores range from 20 to 156. The instrument’s scoring system is determined based on the following three categories: Low satisfaction < 60%, moderate satisfaction 60 – < 80%, and high satisfaction ≥ 80%. The higher the score means the greater the student’s satisfaction according to [34]. In the present study, the overall Cronbach’s alpha for the scale was 0.96, with values for the subscales ranging from 0.94 to 0.98.

### Demographic data of study participants

Demographic information of the students was collected, including age, gender, academic year, and marital status.

### Validity of the tools

To ensure that the contributing students, whose native language is Arabic, clearly understood the study instruments, all items were converted into Arabic. Accordingly, all study instruments were administered in their Arabic validated versions to ensure conceptual, linguistic, and cultural equivalence. Specifically, the Arabic version of the Emotional–Social Intelligence Scale validated by [16] was used in this study. The initial translation was reviewed by a panel of seven university professors fluent in both Arabic and English. Subsequently, a second panel of seven bilingual professors conducted a back-translation of the instruments into English. To further establish content and face validity, five nursing professors specializing in nursing administration carefully appraised the instruments, confirming their appropriateness and validity for use in this study.

To further ensure their suitability for the current population, a pilot study was conducted to assess the instruments’ reliability and clarity. The results revealed satisfactory internal consistency, with Cronbach’s alpha coefficients meeting the recommended threshold of 0.70 proposed by [35], indicating that all scales demonstrated satisfactory internal consistency. In addition, confirmatory factor analysis (CFA) was performed to examine the construct validity of the Arabic versions. All standardized factor loadings were greater than 0.50, and the overall model fit indices indicated an acceptable fit. These results confirm that the adapted tools were valid and reliable for use among Egyptian nursing students.

### Pilot study

A pilot study was carried out to evaluate the clarity and quality of the study materials, the time needed for data collection, and the feasibility, validity, and reliability of the study instruments. It was conducted on 38 students (10% of the sample size) but was not included in the main study sample. The results indicated that no modifications were necessary, as the instruments were clear and appropriate for use.

### Data collection

Data were collected from nursing students between February and April 2025 using structured, self-administered questionnaires. Ethical approval was obtained from the Faculty of Nursing, Zagazig University. The study’s purpose and procedures were clearly explained to students during class sessions, and participation was voluntary. Each questionnaire included a cover letter outlining the study’s aims and assurances of anonymity and confidentiality. To minimize researcher influence and response bias, students completed and submitted the questionnaires independently in a neutral setting.

### Ethical considerations and consent to contribute

This study was conducted following the ethical principles outlined in the Declaration of Helsinki (DoH Oct 2008). The Ethics Committee of the Faculty of Nursing, Zagazig University, established ethical approval under (reference number 379 − 24). All crucial information about the study was presented in the first part of the sheet. The questionnaire encompassed a statement related to the nature and aim of the study. Informed written consent was secured, with participation being voluntary and without academic consequences. Contributors have given their informed consent under the criteria outlined in the Helsinki Declaration. Anonymity and confidentiality were guaranteed, and students were informed, verbally and in writing, that they could withdraw at any time.

### Statistical design

Data analysis was accomplished using AMOS version 24 and IBM SPSS version 27. Descriptive statistics summarized the students’ characteristics. Pearson correlation coefficients were used to determine the power and direction of the bivariate relationships among the study variables. Structural Equation Modeling (SEM) using AMOS was used to evaluate the suggested model, supporting the analysis of complex interactions among the study variables while addressing the measurement errors. SEM is especially useful for testing mediation, as it facilitates the concurrent calculation of both direct and indirect effects, offering deeper insights compared to conventional regression techniques [36]. The statistical significance was measured at a two-tailed p-value < 0.05, with values < 0.01 reflecting high statistical significance.

All path coefficients reported in this study are standardized estimates. Before conducting the SEM analysis, assumptions were assessed and met. Normality was examined through skewness and kurtosis values, which were within the acceptable range of ± 2 [37]. Sampling adequacy was confirmed by a Kaiser Meyer Olkin (KMO) value exceeding 0.80, and the absence of multicollinearity was verified by variance inflation factor (VIF) values below 5 [38]. To test the mediating effects, bootstrapping procedures with 5,000 resamples were conducted using the maximum likelihood estimation method in AMOS. Indirect effects were considered significant when the 95% bias-corrected confidence interval did not include zero. This approach was chosen for its robustness and accuracy in estimating mediation effects. The statistical significance was measured at a two-tailed p-value < 0.05, with values < 0.01 reflecting high statistical significance.

### Results of the study

Table 1 presents the distribution of the studied nursing students’ demographic characteristics. The majority of the studied students (57.6%) were older than 20 years and with a mean age of 20.74 ± 2.41 years. Additionally, 66.1% were female and 89% were single. The distribution of participants across academic years was intentionally balanced according to the stratified sampling design, ensuring proportional representation from each class. The largest proportion was drawn from the second year (25.5%), while the smallest was from the first year (24.4%).

Table 2 specifies the distribution and mean percentage scores of the evaluated variables among nursing students. The results indicate that students had a satisfactory level of emotional–social intelligence (86.3%), with the highest mean percentage score observed in general mood (88.9%), while the adaptability domain had the lowest at 83.6%. Similarly, students specified a high level of self-efficacy (93.5%). The mean percentage score for students’ voice behavior was 61.8%, indicating a moderate level. Within this variable, the promotive voice dimension achieved the highest mean percentage score (63%), indicating a greater tendency among participants to propose constructive ideas and improvements; however, the prohibitive voice dimension was slightly lower (60.6%), suggesting a lesser inclination to raise concerns or highlight risks. Additionally, students revealed a high level of satisfaction (95.3%), with the highest mean percentage score observed in satisfaction with faculty, staff members, support staff, and employees (96.1%), while satisfaction with the physical environment domain had the lowest score (93.3%).

Table 3 studies the correlation analysis among the key study variables. As presented in the table, emotional-social intelligence was found to have significant and positive associations with students’ self-efficacy (*r* = 0.662, *p* < 0.001), voice behavior (*r* = 0.685, *p* < 0.001), and satisfaction (*r* = 0.729, *p* < 0.001). These results suggest that emotional–social intelligence serves as a significant psychological resource, supporting nursing students in achieving academic and social success while engaging in activities that foster both individual and organizational outcomes.

Similarly, self-efficacy is strongly correlated with both voice behavior (*r* = 0.579, *p* < 0.001) and satisfaction (*r* = 0.598, *p* < 0.001), indicating that higher confidence in academic and professional capabilities translates into constructive behavioral expression and greater satisfaction. Moreover, students’ satisfaction is strongly correlated with their voice behavior (*r* = 0.789, *p* < 0.001), indicating that higher student satisfaction constructs a supportive environment that inspires students to participate more actively and express their visions.

**Fig. 3:**
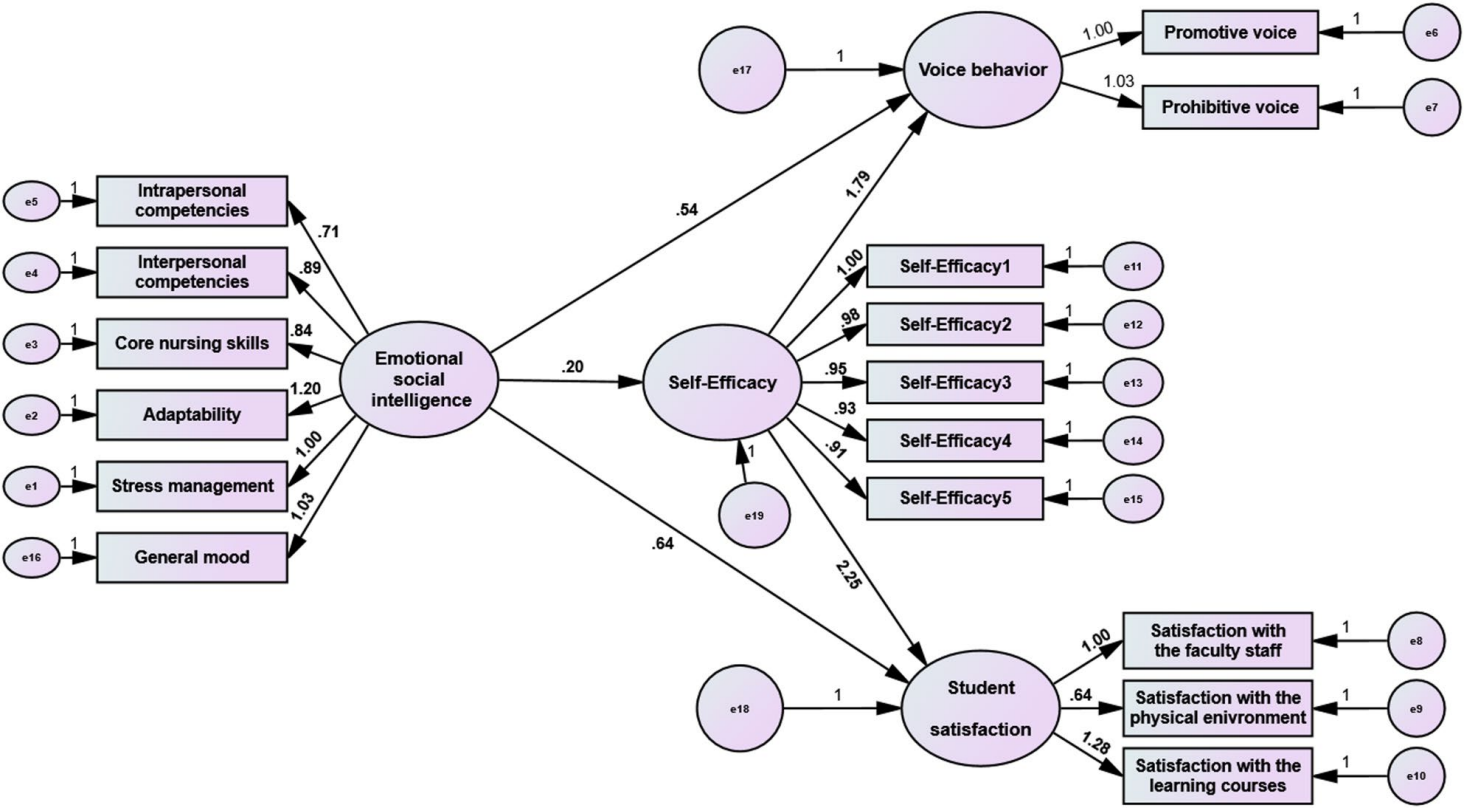
The mediating effect of self-efficacy on the relationship between emotional-social intelligence as regards nursing students’ voice behaviors and their satisfaction (*n* = 381)

Table 4 elucidates the direct and mediating effects, and Fig. 3 outlines the mediating effect of self-efficacy on the relationship between emotional-social intelligence as regards students’ voice behavior and their satisfaction. As exposed, emotional-social intelligence had a significant direct effect on self-efficacy (*β* = 0.20, *p* = 0.001), voice behavior (*β* = 0.54, *p* = 0.001), and satisfaction among the studied students (*β* = 0.64, *p* = 0.001). Similarly, self-advocacy had a significant direct effect on students’ voice behavior (*β* = 1.79, *p* = 0.002) and their satisfaction (*β* = 2.25, *p* = 0.004).

The mediation analysis, employing bias-corrected bootstrapping (5,000 resamples), confirmed significant indirect effects. The analysis revealed that self-efficacy partially mediates the relationship between emotional-social intelligence and voice behavior (Indirect Effect = 0.35, *p* = 0.002, 95% *CI* [0.131/0.505]). This indicates that for every one-unit increase in emotional-social intelligence, voice behavior increases by 0.35 units through the influence of self-efficacy.

Similarly, self-efficacy partially mediated the relationship between emotional-social intelligence and student satisfaction (Indirect Effect = 0.45, *p* = 0.03, 95% *CI* [0.176/0.644]). This specifies that for every one unit increase in emotional–social intelligence, students’ satisfaction is more likely to escalate by 0.45 units indirectly through the influence of their self-efficacy. Since the direct effects of emotional-social intelligence on the outcome variables remained significant even with the mediator present, these results confirm partial rather than full mediation.

Table 5 simplifies the indicators for evaluating model fit in structural equation modeling by comparing the obtained fit indices against the ideal or the acceptable values. Specifically, the Chi-square/df ratio (3.65) falls within the acceptable range (≤ 5), suggesting that the model adequately represents the observed data. The p-value (0.06) is nonsignificant, further supporting the adequacy of the model fit. Additionally, the RMSEA (0.05) reflects a good fit, while the CFI (0.98), IFI (0.96), and TLI (0.97) all exceed the recommended cutoff of 0.95, confirming a strong model fit across incremental indices. The GFI (0.94) also meets the acceptable threshold, reinforcing the model’s robustness. Collectively, these results suggest that the hypothesized model provides a satisfactory and well-fitting representation of the data according to established guidelines [39–42].

**Table 1:** Distribution of the studied nurses’ demographic characteristics (*n* = 381)

Variables	Category	*n*	%
Age (years)	• **≤ 20 years**	162	42.4
	• **> 20 years**	219	**57.6**
Mean age	**20.74 ± 2.41**		
Gender	• **Female**	252	**66.1**
	• **Male**	129	33.9
Academic year of students	• **First year**	93	**24.4**
	• **Second year**	97	**25.5**
	• **Third year**	95	24.9
	• **Fourth year**	96	25.2
Marital status	• **Single**	339	**89.0**
	• **Married**	42	11.0

n = number

**Table 2:** Distribution and analysis of mean percentage values of assessed variables among nursing students (*n* = 381)

Study variables	Maximum score	Mean	±	Standard deviation	% of mean score
Emotional social intelligence components:					
• Intrapersonal competencies	80	70.1	**±**	7.1	87.6%
• Interpersonal competencies	65	57.2	**±**	8.1	88.0%
• Core nursing skills	60	50.3	**±**	6.8	83.8%
• Adaptability	45	37.6	**±**	6.1	83.6%
• Stress management	40	34.1	**±**	6.6	85.3%
• General mood	35	31.1	**±**	3.9	88.9%
Total emotional social intelligence	325	280.4	**±**	26.1	86.3%
Total students’ self-efficacy	20	18.7	**±**	1.4	93.5%
Voice behavior components:					
• Promotive voice	25	15.75	**±**	4.43	63.0%
• Prohibitive voice	25	15.14	**±**	4.24	60.6%
Total voice behavior	50	30.89	**±**	8.58	61.8%
Students’ satisfaction components:					
• Satisfaction with faculty staff members, support staff, and employees	80	76.9	**±**	12.1	96.1%
• Satisfaction with the physical environment	36	33.6	**±**	4.8	93.3%
• Satisfaction with the learning courses used in the faculty	40	38.2	**±**	5.4	95.5%
Total students’ satisfaction	156	148.7	**±**	13.4	95.3%

**Table 3:** Correlation analysis among the key study variables (*n* = 381)

Variables	Emotional social intelligence *r* (*p*-value)	Self-efficacy *r* (*p*-value)	Voice behavior *r* (*p*-value)
Self-efficacy	0.662** (p ˂ 0.001)		
Voice behavior	0.685** (p ˂ 0.001)	0.579** (p ˂ 0.001)	
Satisfaction	0.729** (p ˂ 0.001)	0.598** (p ˂ 0.001)	0.789** (p ˂ 0.001)

r = Pearson correlation. ** Correlation is significant at the 0.01 level (2-tailed)

**Table 4:** The direct and mediated effects as identified in the structural model (*n* = 381)

Effects	β	BC 95% CI Lower/Upper	*P*-value
Direct effects:			
Emotional social intelligence → voice behavior	0.54	0.326/0.962	0.001**
Emotional social intelligence → students’ satisfaction	0.64	0.347/1.502	0.001**
Emotional social intelligence → self-efficacy	0.20	0.175/0.238	0.001**
Self-efficacy → voice behavior	1.79	0.562/2.629	0.002**
Self-efficacy → students’ satisfaction	2.25	1.350/3.422	0.004**
The indirect mediating effects:			
Emotional social intelligence → voice behavior through self-efficacy	0.35	0.131/0.505	0.002**
Emotional social intelligence → students’ satisfaction through self-efficacy	0.45	0.176/0.644	0.03*

Abbreviations: β = Regression coefficient weights. BC = Biased-corrected percentile method.CI = Confidence interval. * (p) Significant level < 0.05. ** (p) Significant level < 0.01

**Table 5:** Indicators for evaluating model fit in structural equation modeling (*n* = 381)

Indices	Acceptable Thresholds	Estimated values	Interpretation	References
χ²/df (Chi-square /degrees of freedom)	• ≤ 5 (acceptable fit) • ≤ 3 (good fit)	3.65	Acceptable model fit	[39, 40]
p-value (for χ²)	• > 0.05 (Nonsignificant)	0.06	Good model fit.	[37, 40]
RMSEA (The Root Mean Square Error of Approximation)	• ≤ 0.08 (acceptable fit) • ≤ 0.06 (good fit)	0.05	Good model fit.	[41]
CFI (Comparative Fit Index)	• ≥ 0.90 (acceptable fit) • ≥ 0.95 (good fit)	0.98	Good model fit.	[41]
IFI (Incremental Fit Index)	• ≥ 0.90 (acceptable fit) • ≥ 0.95 (good fit)	0.96	Good model fit.	[39]
GFI (Goodness of Fit Index)	• ≥ 0.90 (acceptable fit) • ≥ 0.95 (good fit)	0.94	Acceptable model fit	[42]
TLI (Tucker-Lewis Index)	• ≥ 0.90 (acceptable fit) • ≥ 0.95 (good fit)	0.97	Good model fit.	[41]

## Discussion

Emotional–social intelligence is widely recognized as a key determinant of adaptive behavior and effective interpersonal functioning in nursing education. Nursing students with higher levels of ESI demonstrate stronger emotional regulation, perspective taking, and the capacity to build supportive professional relationships, which contribute to enhanced voice behavior and greater satisfaction. Nevertheless, the extent to which ESI translates into such positive outcomes may depend, in part, on students’ perceived self-efficacy, highlighting its potential role as an underlying psychological mechanism [43]. Examining these relationships offers important implications for educational interventions aimed at strengthening both ESI and self-efficacy to promote proactive engagement and satisfaction.

### Emotional-social intelligence and nursing students’ voice behavior

The findings of this study confirm a significant positive relationship between emotional–social intelligence (ESI) and nursing students’ voice behavior, highlighting the role of emotional and interpersonal competencies in fostering proactive communication in educational and clinical settings. This relationship can be explained through Social Exchange Theory, which suggests that students in supportive and emotionally intelligent environments are more likely to engage in constructive behaviors, such as speaking up and sharing ideas [44].

Nursing students with higher levels of ESI tend to demonstrate greater empathy, emotional regulation, and social awareness, enhancing their confidence to express opinions respectfully and constructively [45]. This result aligns with previous studies reporting that emotional intelligence positively influences communication abilities among nurses [46] and is significantly associated with nursing students’ clinical decision-making [47]. Furthermore, it was found that social skills that are a core component of ESI could enhance students’ communication attitudes and willingness to engage in professional dialogue [48].

### Emotional-social intelligence and nursing students’ satisfaction

The present study found that higher levels of emotional-social intelligence (ESI) significantly and directly predicted nursing students’ satisfaction. This outcome can be interpreted through the lens of self-determination theory [49], which suggests that the fulfillment of relatedness, competence, and autonomy needs is fundamental to students’ satisfaction and well-being. Accordingly, nursing students with higher ESI are better equipped to navigate academic and clinical stressors, promote constructive relationships with peers and instructors, and participate proactively in collaborative learning environments. Collectively, these competences strengthen students’ sense of belonging, achievement, and satisfaction [50].

The previous finding is consistent with prior studies showing that ESI correlates with nursing students’ academic performance, satisfaction, achievement, and adaptive learning strategies [51–53, 16]. However [54], who conducted a study of nursing interns, found that ESI did not mediate the relationship between stress and satisfaction, suggesting that contextual factors such as academic workload, year of study, curriculum design, and institutional support may influence this relationship.

### Emotional-social intelligence and nursing students’ self-efficacy

Moreover, the present study demonstrates that ESI has a significant direct and positive effect on nursing students’ self-efficacy. This result is theoretically consistent with Bandura’s [55] social cognitive theory, which posits that students’ beliefs in their capabilities influence their motivation, behavior, and performance outcomes. Harmoniously, this result is consistent with that of [9, 56–60], who reported that emotional intelligence was positively interconnected with nursing students’ self-esteem and self-efficacy both directly and indirectly by shaping students’ coping styles.

While most studies indicate a positive relationship, some research suggests contextual variability; for instance, stress levels, curriculum design, and cultural factors may moderate the effect of ESI on students’ self-efficacy [54, 61]. This emphasizes the need to interpret the findings within specific educational and cultural environments.

### The mediating effect of self-efficacy on the relationship between emotional-social intelligence and students’ voice behavior

Furthermore, the existing study results confirmed that the relationship between emotional-social intelligence and nursing students’ voice behavior is partially mediated by their self-efficacy, indicating that although emotional intelligence independently impacts students’ preparedness to engage in open communication, self-efficacy augments and simplifies the interpretation of emotional aptitudes into actual behavioral outcomes.

Consistently [62], revealed that promoting emotional intelligence and self-efficacy may escalate nursing students’ competencies in clinical decision-making. Compatibly [63], mentioned that self-efficacy played an arbitrator role between emotional intelligence and clinical communication commitment. Nevertheless [61], indicated that in some contexts, high emotional intelligence does not automatically translate into positive behaviors unless supported by confidence, skill mastery, and institutional encouragement. This emphasizes the importance of contextual factors in nursing education and underscores the value of targeted interventions to develop both ESI and self-efficacy.

### The mediating effect of self-efficacy on the relationship between emotional-social intelligence and students’ satisfaction

Finally, our study findings revealed that self-efficacy partially mediates the relationship between ESI and nursing students’ satisfaction, indicating that while ESI directly enhances students’ satisfaction with their academic and clinical experiences, self-efficacy strengthens and facilitates the translation of emotional social competencies into a more positive and fulfilling educational experience.

Additionally [64], found a partial mediating effect of self-efficacy in the relationship between emotional intelligence and nursing students’ clinical performance. Correspondingly [65], indicated that self-efficacy mediated the connection between emotional intelligence and job satisfaction in healthcare professionals. Likewise [66], reported that self-efficacy mediated the relationship between emotional intelligence and academic engagement among university students [67]. Also, support these results. Conversely, the impact of ESI on satisfaction may be weaker when self-efficacy is low or when students face high levels of external stressors [61].

### Limitations and future directions

Although this study affords valuable insights, it is essential to identify certain limitations. First, as it was carried out within only one institution, the findings may not be generalizable to other nursing faculties or cultural settings. Second, the cross-sectional scheme limits the ability to create causal effects. Finally, the dependence on self-reported assessment may have presented response bias, particularly social desirability. Future research should consider experimental or longitudinal designs to elucidate the causal pathways, repeat the study through miscellaneous settings and populations to improve generalizability, and integrate multi-source or objective procedures to reinforce validity.

## Conclusion

Our study determines that emotional-social intelligence plays an important role in improving nursing students’ voice behavior and their overall satisfaction. Outstandingly, self-efficacy was recognized as a crucial mediating factor, emphasizing that students who have greater emotional-social intelligence are more assertive in expressing their concerns and ideas, which in turn positively encourages their clinical experiences and satisfaction with their learning. These findings highlight the importance of incorporating emotional-social intelligence improvement into nursing education programs to raise professional growth, proactive communication, and better academic and clinical satisfaction among students.

### Implications for nursing management and nurse educators

#### Implications for nursing management

First, they should create supportive environments through establishing open communication channels and feedback schemes that enable nursing students to explicit their suggestions and concerns without fear of negative consequences. Second, developing structured mentorship programs by pairing nursing students with proficient nurses who can monitor them in implementing emotional-social intelligence in clinical practice and decision-making. Third, rewarding and recognizing students’ voice contributions in patient safety or care deliberations to strengthen the value of speaking up. Finally, they should encourage confidence and wellness through introducing innovations or workshops that enhance resilience and self-efficacy to help students manage their clinical stress more effectively.

Implications for nurse educators: First, they should incorporate ESI training modules, including emotional regulation workshops, role-playing exercises, and empathy-building activities, to help students manage stress and communicate effectively in clinical and academic settings, to strengthen students’ emotional-social intelligence. Second, employ mentorship programs, guided reflective practices, and simulation-based clinical exercises that allow students to practice skills in a safe environment, reinforcing their belief in their capabilities to increase students’ self-efficacy and their problem-solving skills. Third, they should encourage students’ active participation through incorporating peer feedback, group discussions, and student-led projects to foster their voice behavior in the academic settings. Finally, providing regular constructive feedback on clinical performance and interpersonal skills, encouraging students to self-assess and set achievable goals, which can strengthen self-efficacy and support their satisfaction with the learning experiences.

## Data Availability

The data that support the findings of this study are available from the corresponding author upon reasonable request.
